# The *CARD8* p.C10X mutation associates with a low anti-glycans antibody response in patients with Crohn’s disease

**DOI:** 10.1186/1471-2350-14-35

**Published:** 2013-03-18

**Authors:** Francis Vasseur, Boualem Sendid, Franck Broly, Corinne Gower-Rousseau, Aurore Sarazin, Annie Standaert-Vitse, Jean-Frederic Colombel, Daniel Poulain, Thierry Jouault

**Affiliations:** 1 Université Lille Nord de France, Lille, 59000, France; 2 UDSL, Lille, 59000, France; 3 Inserm U995, Team 2, Faculté de Médecine H. Warembourg, Pôle Recherche, Place Verdun, Lille, F-59000, France; 4 EA2694, Lille, 59000, France; 5 Pôle de Santé Publique, Lille, 59000, France; 6 Service des Maladies de l’Appareil Digestif et de la Nutrition, Lille, 59000, France; 7 Service de Parasitologie Mycologie, Institut de Microbiologie, Lille, 59000, France; 8 EA2679, Lille, 59000, France; 9 CHRU Lille, Lille, 59000, France

**Keywords:** Crohn's disease, Anti-glycan antibodies, CARD8/TUCAN, ASCA/ALCA, Inflammasome, Adaptive immunity

## Abstract

**Background:**

Crohn’s disease (CD) is associated with elevated anti-glycans antibody response in 60% of CD patients, and 25% of healthy first-degree relatives (HFDRs), suggesting a genetic influence for this humoral response. In mice, anti-glucan antibody response depends on the NLRP3 inflammasome. Here, we explored the effect of mutated *CARD8*, a component of the inflammasome, on anti-glycans antibody response in human.

**Methods:**

The association between p.C10X mutation (rs2043211) of the *CARD8* gene and the levels of anti-glycans antibody response was examined in 39 CD families. The family-based QTDT association test was used to test for the genetic association between *CARD8* p.C10X mutation and anti-glycan antibodies in the pedigrees. The difference in antibody responses determined by ELISA was tested in a subgroup of CD probands (one per family) and in a subgroup of HFDRs using the Wilcoxon Kruskal Wallis non-parametric test.

**Results:**

The QTDT familial transmission tests showed that the p.C10X mutation of *CARD8* was significantly associated with lower levels of antibody to mannans and glucans but not chitin (p=0.024, p=0.0028 and p=0.577, for ASCA, ALCA and ACCA, respectively). These associations were independent of *NOD2* and *NOD1* genetic backgrounds. The p.C10X mutation significantly associated or displayed a trend toward lower ASCA and ALCA levels (p=0.038 and p=0.08, respectively) only in the subgroup of CD probands. Such associations were not significant for ACCA levels in both subgroups of CD probands and of HFDRs.

**Conclusion:**

Our results show that ASCA and ALCA but not ACCA levels are under the influence of *CARD8* genotype. Alteration of CARD8, a component of inflammasome, is associated with lower levels of antibodies directed to mannans and glucans at least in CD patients.

## Background

In humans, the antibody response to glycans has been associated with various infectious and autoimmune diseases [1]. Regarding yeast glycans, monitoring of the anti-mannan antibody response is used for the diagnosis of invasive *C*. *albicans* infections [2]. Other anti-mannan antibodies such as anti-*S*. *cerevisiae* antibodies (ASCA) are associated with Crohn’s disease (CD) where they represent the most frequently found serological marker [3] and at this time, the most related markers for early CD diagnosis [4]. ASCA levels are elevated in 60% of CD patients and in 25% of their healthy first-degree relatives (HFDRs) [5-7]. Although still unknown, one possible origin for this antibody response would be an abnormal adaptive response to the pathogenic yeast *C*. *albicans*[8]. As well as ASCA, other specific serological markers for CD have been described including anti-glucan (ALCA) and anti-chitin (ACCA) antibodies [9], all of them being generated during invasive *C*. *albicans* infection [10].

In this paper, we were interested in the role of caspase activating and recruitment domain 8, CARD8, also known as CARDINAL or TUCAN (tumor-up-regulated CARD-containing antagonist of caspase 9) in the regulation of anti-glycans antibodies response. CARD8 is a 48-kDa peptide predominantly expressed in monocytes, placenta, lymph nodes, and spleen. The *CARD8* gene is located at 19q13.3 between *rs736289* and *rs281379* loci recently associated with CD in a GWAS meta-analysis [11]. CARD8 has structural similarity with NOD1 (CARD4) and NOD2 (CARD15), whose mutations are well known risk factors for CD [12]. CARD8 protein functions as an inhibitor of apoptosis, by blocking procaspase 9, as well as an inhibitor of NF-κB activation [13] and it is a component of NLRP3 inflammasome [14]. NLRP3 inflammasome (formerly cryopyrin, CIAS1, and NALP3) is the best characterized inflammasome complex. It includes ASC (apoptosis-associated speck-like protein), caspase 1 and CARD8 [14,15]. NLRP3 inflammasome is activated by many microbial stimuli and by endogenous danger signals such as ATP and monosodium urate [15]. Other activators include indigestible particulates like silica and alum [16] but also fungal pathogens such as *C*. *albicans*[17-20].

Assembly of inflammasome proteins enables activation of caspase 1 and, thereby, initiates the second danger signal leading to the cleavage of the inflammatory cytokine IL-1β into its biologically active form. IL-1β is involved in animal models of fungal infection, together with IL1-α and IL-18 [21-25]. However, there are now strong evidences that it has a significant role in modulating the adaptive immune response [26,27]. Indeed, B-cells are directly activated by β-glucans through NLRP3, suggesting a critical role of B-cell-intrinsic NLRP3 for anti-glycans antibody responses [19]. NLRP3 inflammasome is thus considered to be a critical component for regulating β-glucan-induced innate [19,28], but also adaptive immune responses in mice [19]. In humans, multiple NLRs are expressed in peripheral B lymphocytes, notably NOD1, NOD2, NLRP1 and NLRP3 [29]. *NOD2* and *NOD1* genetic variants together with their relationship with genetic susceptibility to Crohn's disease have been reported to be associated with anti-glycan antibody levels [30,31].

The deleterious mutation of *CARD8* (p.C10X) predicting a stop codon at position 10, prematurely terminates the protein. This mutation has consequences for the protein’s function in both inflammasome-mediated processes and NF-κB suppression. Several studies have thus concerned the influence of the p.C10X variant of *CARD8* on the genetic risk of chronic inflammatory diseases particularly CD. However, the results remain controversial [32-36].

Given the participation of CARD8 in the NLRP3 inflammasome complex, the role of NLRP3 inflammasome in the antibody response to yeast glycans, the structural similarities displayed by the proteins encoded by the *CARD8*, *NOD2*, *NOD1* genes and their involvement in related pathways that modulate activation of immune cells and inflammation, the aim of this study was thus to investigate the relation between the p.C10X mutation of the *CARD8* gene and antibody response to yeast glycans. As the p.C10X mutation was investigated in view of the antibody response to glycans in humans, we took advantage of the well-characterized antibody response to yeast glycans observed in families from Northern France with a strong aggregation of CD cases.

## Methods

### Patients and study design

CD families were recruited from the EPIMAD Registry [37]. Diagnosis of CD was based on the usual criteria, and phenotypes were defined according to the Montreal classification [38]. A peripheral venous blood sample was obtained from each participant at time of recruitment. The study protocol was approved by the ethics committee of the University Hospital of Lille, and informed written consent was obtained from all study participants.

ASCA, ALCA and ACCA levels and genotypes at rs2043211 were obtained for 200 subjects (87 men and 113 women) from 39 CD pedigrees, among them 76 subjects were affected with CD. A subgroup of one CD proband per family and a subgroup of 39 HFDRs were constituted. When several HFDRs were eligible in a family, one HFDR was selected at random. A previous reported panel of control families was used to determine the allelic frequency of rs2043211 in a control population from the same geographic area [30].

### Genotyping

Genotyping for rs2043211, which consists of a T to A transversion (c.30A > T) located at the third nucleotide of codon 10 of *CARD8*, generating a premature stop codon (p.C10X) and a severely truncated CARD8 protein, was performed by PCR-RFLP. Briefly, DNA was extracted from whole blood using a QIAamp DNA blood kit (Qiagen, Valencia, CA, USA) and amplified with the forward primer 5'-GAGACAGAGGCAGAGCCATT-3' and reverse primer 5'-CCCCTGAGTTCGATGAAAAA-3'. The amplified fragment (175 bp) was then digested with DpnI (New England Biolabs, Ipswich, MA, USA), which recognizes the A allele, generating two fragments 150 and 25 bp in size. The digestion products were run on a 4% agarose gel. Ten DNA samples were also sequenced with the same primers used for amplification, to verify the polymorphism. Genotypes at NOD2 (R702W, G908R, 1007fs insC) and at the NOD1 +32656 loci were determined using PCR-RFLP as previously described [30].

### Detection of anti-yeast glycan antibodies

All sera were assayed using a panel of tests that detect ASCA, ALCA and ACCA (IBDX; Glycominds, Lod, Israel) [9]. This panel consists of kits involving three antigens: *S*. *cerevisiae* mannan, laminaribioside and chitobioside for the detection of ASCA, ALCA and ACCA, respectively. Para-nitrophenyl derivatives of each antigen were covalently bound to the surface of microtiter wells using a linking agent (oligomer of 1,8-diamino-3,6-dioxaoctan; Sigma Chemical Co., St. Louis, MO, USA). These tests were performed according to the manufacturer’s instructions. Antibody levels were expressed in arbitrary units (AU), relative to the manufacturer’s calibrators. The antibody titers for each sample were calculated by dividing the average optical density (OD) of the sample by the average OD of the calibrator, multiplied by the number of units denoted by the calibrator tube label. Individuals were declared positive when levels of antibody in their sera were superior to 50, 60 and 90 AU for ASCA, ALCA and ACCA, respectively.

### Statistical analyses

Fitting the Hardy-Weinberg equilibrium, allelic frequencies and association with the disease were determined with the Haploview software [39]. The family-based association test implemented in the QTDT software [40] available at (http://www.sph.umich.edu/csg/abecasis/QTDT/index.html) was used to test for a genetic association between the p.C10X mutation and a quantitative trait in the 39 pedigrees. In QTDT analyses, empirical robust *p* values were obtained following 10,000 Monte Carlo permutations. The CLUMP software [41] was used to compare allele frequencies. As a classical Chi^2^ test the CLUMP software allows comparison of frequencies between groups but a robust significance is assessed using repeated Monte Carlo permutations. Moreover, from the original 2 × 4 table (for the 4 groups of subjects) the CLUMP software clumps columns together in a new 2 × 2 table seeking to maximize Chi^2^ value allowing assessment of post-hoc tests whose robust significance is obtained using Monte Carlo permutations. All CLUMP analyses were performed following 100 000 Monte Carlo permutations. When statistical investigations were not possible with the family-based association test implemented in the QTDT software, as there were families with more than one member affected with Crohn's disease, for a balanced influence of these families in "classical" statistical analyses, a subgroup of one CD proband per familiy was selected. Likewise for healthy relatives, a panel of one healthy relative selected at random in each family was established. Comparison of antibody responses was performed in the subgroups of CD probands and of HFDRs with the Wilcoxon Kruskal Wallis non-parametric test. The Chi2 test was used to compare the proportions of anti glycan positive subjects between CD patients and healthy relative subjects (HFDR). All "classical" statistical procedures were conducted with the JMP Pro 9.02 software (SAS Institute, Cary, NC).

## Results

### Distribution of the CARD8 p.C10X mutation (rs2043211) in the CD families and in control populations

Allelic frequencies were not significantly different between the CD patients, the unaffected subjects (p=0.89) of the 39 CD families, the subjects from French control families and from the CEU panel of the HapMap version 3 release R2 (Table 1), but displayed a suggestive trend of association as analyzed by the CLUMP software (p=0.09). Post hoc tests disclosed that the chi2 was maximal when clumping subjects from French control families and HapMap subjects versus all subjects from our CD families: CD patients and unaffected subjects (p=0.06). There was a trend toward a lower frequency of the *CARD8* p.C10X mutation in the population from the CD families as compared with reference (French control families and HapMap CEU) Caucasian populations (Table 1). However there was no association nor trend of association between the p.C10X *CARD8* mutation and localization (L1, L2, L3, L4) and behavior (B1, B2, B3) of the disease according to the Montreal classification (data not shown).

**Table 1:** Allelic frequencies of the p.C10X mutation in the populations under study

		**p.C10X MAF**	**95% CI**	**Comparison between the 4 groups (dof=3)**	**Comparison (dof=1)**	**Comparison (dof=1)**
1	CD patients	0.243	[0.183-0.316]	p = 0.09	1 vs. 2	1+2 vs. 3+4
2	Healthy subjects from CD families	0.238	[0.177-0.309]			
					p=0.89	
						p = 0.06
3	French control families	0.347	[0.251-0.471]		3 vs. 4	
					p = 0.53	
4	HapMap CEU	0.315	[0.271-0.361]			

Frequencies are those determined in pedigrees using the Haploview software. All frequencies comparisons were performed with the CLUMP software and robust p values were obtained following 100,000 Monte Carlo permutations. (MAF: minor allele frequency, dof: degree of freedom).

As anti-glycan antibody levels are well known to be significantly higher in CD patients as compared to healthy subjects, all analyses were adjusted according to the affected/non-affected status.

### CARD8 p.C10X mutation is significantly negatively associated with ASCA levels in CD families

In familial transmission tests (QTDT) adjusted by the CD status, including the whole population (n=200) the p.C10X *CARD8* mutation was significantly associated with a lower ASCA level (p=0.024). As we previously reported that ASCA levels were associated with the genotypes at the NOD2 (R702W, G908R, 1007fs insC) and at the *NOD1* +32656 loci [30], QTDT analyses we adjusted according to the genotypes at the *NOD2* and *NOD1* loci. Thus ASCA levels remained significantly associated with the *CARD8* p.C10X mutation (p=0.023). Similar QTDT results were obtained in CD families for the association between the CARD8 p.C10X mutation and the binary trait ASCA positive or negative according to the 50 units threshold (Table 2).

**Table 2:** **Results of familial QTDT analyses including the whole population (n=200**)

	**Adjusted by the CD status**		**Adjusted by the CD status, and *NOD2 *and *NOD1 *genotypes**	
	**p value**	**Z for mutated allele**	**p value**	**Z for mutated allele**
ASCA level	0.024	−24.59	0.023	−24.58
ASCA binary trait (positive/negative)	0.05	−0.146	0.05	−0.145
ALCA level	0.0035	−17.08	0.0034	−17.17
ALCA binary trait (positive/negative)	0.0025	−0.232	0.0026	−0.230

The Z values reflect the strength of associations; negatives values reflect an association with a lower level of the trait under study. Positive values would reflect association with a higher level of the trait. *NOD2*, (R702W, G908R, 1007fs insC mutations) and the *NOD1* +32656 genotypes were from Vasseur et al. [30].

**Figure 1:**
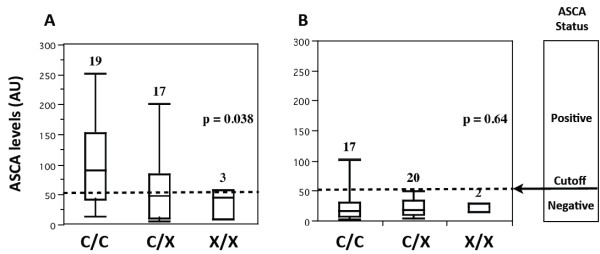
**Measurement of anti**-**mannan antibody levels in human sera according to p.C10X genotype.** Anti-mannan antibody (ASCA) levels were determined in sera from CD probands (**A**) and healthy first-degree relatives (**B**) by ELISA. Antibody titers (expressed in AU according to the reactivity of the manufacturer’s calibrator) in samples from subjects who were wild-type (C/C), heterozygous (C/X), or homozygous (X/X) for the p.C10X mutation. Positive or negative ASCA status was determined according to the cutoff value of 50 AU determined according to the manufacturer's instruction.

In the subgroup of 39 CD probands, ASCA levels were significantly lower according to the p.C10X genotype (p=0.038, Figure 1A) in agreement with the above reported familial QTDT analyses. In contrast, no significant association could be detected between the p.C10X *CARD8* mutation and ASCA level in the subgroup of 39 HFDRs (p=0.64, Figure 1B).

### CARD8 p.C10X mutation is significantly negatively associated with ALCA levels in CD families

The results observed for anti-mannan antibodies (ASCA) levels prompted us to perform similar analyses on the anti-glucan antibodies (ALCA), known to be associated with CD status and whose production was dependent on inflammasome activation. In familial transmission tests (QTDT) adjusted by the CD status, including the whole population (n=200), the p.C10X *CARD8* mutation was significantly associated with a lower ALCA level (p=0.0035). Likewise this association remained largely significant following adjustment by the *NOD2*, *NOD1* genotypes (p=0.0034). Similar QTDT results were obtained in CD families for the association between the *CARD8* p.C10X mutation and the binary trait ALCA positive or negative according to the 60 units threshold (Table 2).

**Figure 2:**
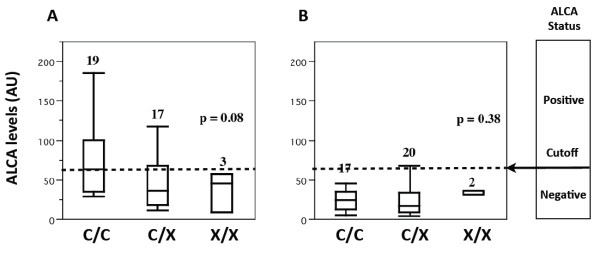
**Measurement of anti**-**glucan antibody levels in human sera according to p.C10X genotype.** Anti-glucan antibody (ALCA) levels were determined in sera from CD probands (**A**) and healthy first-degree relatives (**B**) by ELISA. Antibody titers (expressed in AU according to the reactivity of the manufacturer’s calibrator) in samples from subjects who were wild-type (C/C), heterozygous (C/X), or homozygous (X/X) for the p.C10X mutation. Positive or negative ALCA status was determined according to the cutoff value of 60 AU determined according to the manufacturer's instruction.

In the subgroup of 39 CD probands there was a trend toward a lower ALCA level according to the p.C10X genotype (p=0.08, Figure 2A). A similar analysis in the subgroup of 39 HFDR, did not detect a significant association (p=0.38, Figure 2B).

### ACCA levels are independent of CARD8 p.C10X mutation

In familial transmission tests (QTDT) adjusted by the CD status including the whole population (n=200), no association was observed between the p.C10X *CARD8* mutation and the ACCA levels (p=0.577). Similarly, no associations were detected in the subgroups of 39 CD probands and of 39 HFDR (p=0.95 and p=0.22, respectively).

### Distribution of anti-glycans antibody responders in the population examined

Considering the subgroup of one CD proband per pedigree and the subgroup of HFDRs, anti-glycans antibody levels (ASCA, ALCA) were elevated in CD probands as compared with the group of healthy first-degree relatives (HFDRs) in agreement with previously reported data [5,6]: ASCA levels were 78.7±77.6 for CD probands and 24.1±27.0 for HFDRs (p<0.0001), ALCA levels 64.1±56.1 for CD probands and 26.4±20.0 for HFDRs (p=0.0002). The proportion of anti-glycans antibody positive subjects (Table 3) was in agreement with the data from the literature [5,6].

**Table 3:** **Proportion of anti**-**glycans antibody positive subjects among the CD probands and the HFDRs**

	**ASCA positive**				**ALCA positive**				**ASCA or ALCA positive**			
	**n**	**%**	**95% CI**	**p value**	**n**	**%**	**95% CI**	**p value**	**n**	**%**	**95% CI**	**p value**
CD probands (n=39)	21	53.8	[38.6-68.4]	p<0.0001	15	38.4	[24.9-54.1]	p=0.0008	23	58.9	[43.4-72.9]	p<0.0001
HFDR (n=39)	4	10.3	[4.1-23.6]		3	7.7	[2.7-20.3]		6	15.4	[7.3-29.7]	

ASCA positivity was according to the 50 AU threshold and ALCA positivity according to the 60 AU threshold.

In the 39 CD probands subgroup there were less ASCA positive patients (45.0%) in those having at least one p.C10X *CARD8* mutation (dominant model) than in those being wild-type at this locus (63.2%). Although this analysis on a limited number of subjects did not reach significance (p=0.2) it was in agreement with familial association results. Likewise in the subgroup of 39 CD probands the ALCA positive CD patients were less frequent (25.0%) in those with at least one p.C10X *CARD8* mutation than in those who were wild-type (52.6%) but this difference did not reach significance (p=0.07). The proportion of CD patients being either ASCA or ALCA positive were 50.0% in those having at least one p.C10X *CARD8* mutation versus 68.4% in the wild type ones (p=0.2).

## Discussion

This study investigated the relationships between a mutation of the *CARD8* gene encoding a component of inflammasome and antibody response in humans in the context of Crohn’s disease (CD) where anti-yeast glycan antibodies are well documented. Our results show that decreased levels of antibody to mannan (ASCA) and Glucans (ALCA) but not chitin (ACCA) are associated with the *CARD8* p.C10X mutation. These results show that the *CARD8* p.C10X mutation, despite not being associated with Crohn's disease *per se* (at least in French Caucasians) is associated with some but not all anti-yeast glycan antibody levels (ASCA and ALCA but not ACCA) in CD families.

Among several serological markers of CD, the anti-glycan response has been shown to be one of the main features of this disease [4,42]. The origin of this response is still unclear although the possible involvement of *C*. *albicans* has been proposed [8] and a link has been shown between *Candida* colonization and ASCA [43]. Although high ASCA levels are considered to be markers of CD (60% of CD patients are highly positive for ASCA), it is unclear why some CD patients do not have elevated ASCA levels independently of the gravity of the illness or of its location.

Compelling evidence have led to the consensus that CD is a multifactorial disorder that results from an uncontrolled inflammatory response to endoluminal microorganisms in genetically predisposed individuals. To date, over 70 distinct genomic loci have been shown to confer susceptibility for CD [11]. Most of them encode or are located in the vicinity of genes that are involved in the inflammation process, the modulation of host-microbe interactions and in the maintenance of epithelial homeostasis in the gut. The most strongly association with CD has been shown for the gene coding the innate pattern recognition receptor *NOD2*/*CARD15*[12]. However, other genes such as *CARD8*, located at an IBD locus, have also been shown to be involved in CD [35,36].

CARD8 is a protein whose function has not been fully elucidated. It is involved in regulation of pro inflammatory response involving NFκB [13] and was therefore proposed as a susceptibility gene for CD [34]. However, the association between *CARD8* and CD remains controversial [44,45] although the influence of NFκB in ASCA production has been reported [46]. CARD8 also participates to the formation of inflammasome NLRP3 [14,15] involved in innate immunity, thus impacting the cellular response based on caspase-1 and NFκB [47]. Using NLRP3 knockout mice, inflammasome has been shown to be important in production by phagocytic cells of IL-1β [19], a cytokine important in inflammatory processes and involved in the modulation of the adaptive immunity [48,49]. NLRP3 also plays a role in the adaptive immunity since it is implied in B-cell activation leading to production of anti-glycans antibody, in a manner independent of the innate immunity [19].

In agreement with the literature [44], our current results showed that the *CARD8* mutation was not associated with CD in our panel of French families. On the other hand, the p.C10X allele was associated with a significantly lower antibody response with respect to glycans which are present on the surface of yeasts. Surprisingly, such association was not evidenced for antibody response directed against chitin, a wide spread component, highlighting a difference in either antigen presentation or regulation according to glycans. This is in accordance with the observation that B-cells from NLRP3 knockout mice are unable to produce antibodies after stimulation by glucans [19] suggesting that production of anti-glucan antibodies by B-cells is at least partly dependent on the activity of inflammasome. Although the putative biological link between the p.C10X mutation and the anti-glycans antibody response remains to be clarified these results represent the first clue as to a relationship between innate and adaptive immunity in CD.

B-cells may be directly activated by glycans [50]. However, B-cell may also be activated by neutrophils through the production of the B-cell specific cytokine (BAFF) allowing natural immunoglobulins production by B-cells [51,52]. We previously reported that +32656 *NOD1* insertion-deletion polymorphism associates with unresponsiveness towards glycans [30]. NOD1/CARD4 is an intracellular sensor for peptidoglycan from Gram-negative bacteria that plays an important role in neutrophil function, recruitment and activation [53]. Considering the role that NOD1 plays on neutrophils activation, which in turn may result on the alteration of B-cells activation and antibody production, we thus examined the possible relation between *NOD1* polymorphism and *CARD8* p.C10X mutation toward anti-glycans antibodies production. *CARD8* p.C10X mutation and +32656 *NOD1* insertion-deletion polymorphism did not show any additive participation to anti-glycans modulation and *CARD8* mutation by itself associated with antibody production phenotype. Thus our observations suggest that *CARD8* and *NOD1* genetic variants independently associate with antibody levels through independent pathways without any detected additivity.

HFDRs analyzed separately did not show any significant association between ASCA, ALCA or ACCA and the *CARD8* p.C10X mutation. However we cannot exclude that the low antibody levels observed in HFDRs make difficult the detection of a significant difference according to genotype, especially with a limited subgroup of subjects.

Severity of CD is associated with higher ASCA and ALCA levels [54,55]. This suggests a possible involvement of inflammasome in: (i) the production of pro-inflammatory cytokines such as IL-1β and secondarily IL-17 leading to the development of the CD inflammatory process [56,57]; and (ii) an effective anti-glycan response [19]. CARD8 has been shown to regulate NFκB-dependent cell activities, it is thus anticipated that the absence of active CARD8, evidenced by an altered ASCA response, could be associated with a more favorable prognosis in terms of disease evolution. Here, we were unable to detect a significant association between the genetic alteration of *CARD8* and the severity and location of the disease according to the Montreal classification [38].

## Conclusions

The main result of our study is that decreased levels of anti-yeast glycan antibody levels are associated with the *CARD8* p.C10X mutation in the families under investigation. Associations may be considered as confident as they were based on QTDT analyses including the whole population under study (200 subjects). Regarding the stratifyed analyses in the subgroup of 39 patients presenting Crohn's disease and of 39 healthy relatives, results may be interpreted with caution regarding the low number (39) of subjects included in the analyses. It is likely that CD affected patients have a major weight in the QTDT familial analyses and that the lack of anti glycan antibody difference association with *CARD8* p.C10X in the healthy relatives subgroup may reflect a lack of statistical power. Despite not being directly associated with Crohn's disease (at least in French Caucasians) the *CARD8* p.C10X mutation, which is thought to affect regulation of inflammasome activity, is specifically associated with some anti-yeast glycan antibody levels (ASCA and ALCA) in CD families. Confirmation of a mechanistic role of CARD8 in anti-glycan antibody production may be important in better understanding the participation of inflammasome in adaptive response in particular against yeast components, but also for individual diagnosis in the case of patients presenting *CARD8* mutation for whom ASCA levels would be low whatever the gravity of their disease.

## Competing interests

The authors declare that they have no competing interests.

## Authors’ contributions

FV: carried out the studies and data analyses (statistics and genetics), and wrote the manuscript. BS: carried out the studies and data analyses (antibody response), participated to draft the manuscript. FB: participated to the genotyping. CGR: collected and organized the samples and helped to draft the manuscript. AS: carried out the genotyping. AVS: organized the samples and helped to draft the manuscript. DP: helped to draft the manuscript. JFC: helped to draft the manuscript. TJ: coordinated the study and wrote the manuscript. All authors read and approved the final manuscript.

## Pre-publication history

The pre-publication history for this paper can be accessed here:

http://www.biomedcentral.com/1471-2350/14/35/prepub
